# Complete mitochondrial genome sequence of neotropical fish *Astyanax giton* Eigenmann 1908 (Ostariophysi; Characidae)

**DOI:** 10.1080/23802359.2017.1403869

**Published:** 2017-11-25

**Authors:** Cynthia Aparecida Valiati Barreto, Manuela Maria Cavalcante Granja, Pedro Marcus Pereira Vidigal, Anderson Oliveira Carmo, Jorge Abdala Dergam

**Affiliations:** a Laboratório de Sistemática Molecular- Beagle, Departamento de Biologia Animal, Universidade Federal de Viçosa, Viçosa, Minas Gerais, Brazil;; b Núcleo de Análise de Biomoléculas (NuBioMol), Centro de Ciências Biológicas (CCB), Universidade Federal de Viçosa, Viçosa, Minas Gerais, Brazil;; c Departamento de Biologia Geral, Instituto de Ciências Biológicas, Universidade Federal de Minas Gerais, Belo Horizonte, Brazil

**Keywords:** Freshwater fish, mitogenome, next-generation sequencing

## Abstract

Here we report, for the first time, the complete mitochondrial genome of *Astyanax giton* which, together with other species, are popularly known as tetras. The mitogenome’s length is 16,643 bp, containing 13 protein-encoding genes (CDS), two ribosomal RNAs (rRNA), 22 RNA transfer (tRNA) and one control region (D-loop). As for other vertebrates, all genes are encoded on the heavy strand except for *ND6* and eight *tRNA* genes. In the phylogenetic analyses, this species and other *Astyanax* were paraphyletic.

In the Characidae family, the genus *Astyanax* is a taxon that encompasses species with poorly known phylogenetic kinships, popularly known as tetras. It currently comprises 146 valid species with a wide distribution on the Neotropical region (Lima et al. 2003), occurring from the southern United States to northern Argentina (Froese and Pauly 2011). The taxonomy of the *Astyanax* genus is still unsettled, although morphological (Mirande 2009) and molecular data (Javonillo et al. 2010) indicate that this taxon is paraphyletic. Until the present date, only two mitogenomes of the genus *Astyanax* have been described, which underlines the relevance of the description of the complete mitogenome of *Astyanax giton*.

*Astyanax giton* was collected in the Doce River Basin, at the headwaters of the Latão Stream, in the city of Coimbra (20°49′66″S, 42°49′58″W) in the state of Minas Gerais, in southeastern Brazil. The voucher specimen was deposited in the scientific collection of the Museum of Zoology João Moojen, at the Federal University of Viçosa, Minas Gerais, Brazil (voucher no. CT2205). This work was carried out with DNA purified from the muscle tissue and the genomic library was sequenced with 2 × 300 bp *paired-end* reads using the Illumina MiSeq (Illumina Inc., San Diego, CA). The quality of the sequencing was evaluated using FASTQC v.0.11.5 and the *reads* were trimmed (Q20 score) and filtered by size (75 nt) using Trimmomatic v0.33 (Bolger et al. 2014). The mitogenome of *A. giton* was assembled using a *de novo assembly* in CLC Genomics Workbench v6.5.1 (CLC Bio, Boston, MA). The mitogenome sequence was selected and annotated using MitoAnnotator (Iwasaki et al. 2013).

The mitogenome of *A. giton* is typical of vertebrates (Boore 1999) and its length was 16,643 bp (GenBank access no. MF805815), with a 121-fold coverage. Thirty-seven genes were functionally annotated, 13 coding DNA sequences (CDS), two ribosomal RNA (*rRNA*), 22 transfer RNAs (tRNA*)* and a control region (D-loop) of 997 bp. Most of the genes are located on the heavy strand (H), except for eight *tRNAs* and *ND6*. Besides the D-loop region, *A. giton* has 14 intergenic regions in its mitogenome, which add up to 94 bp in length.

The 13 CDS of *A. giton* represent 68.6% of the total mitogenome. The *COI* and *ND3* genes start with the GTG codon, whereas others show usual ATG codon. Three of the 13 CDS contain the TAA stop codon (*ND1*, *ND4L* and *ND6*); five the incomplete T- stop codon (*COII*, *ATPase6, COIII*, *ND3* and *ND4*); three the TAG stop codon (*ND2*, *ATPase8* and *CYTB*) and finally, two had the AGG stop codon (*COI* and *ND5*).

Mitogenomes of the Characidae species available at NCBI were aligned and the phylogeny reconstruction was done using the Bayesian inference (BI) and maximum-likelihood (ML) methods, using the GTR + I + G model, with *Acestrorhynchus* sp. as outgroup (Figure 1). In the phylogenetic analysis, the genus *Astyanax* was considered paraphyletic, with *A. giton* as a sister group of ((*Astyanax paranae*, *Astyanax mexicanus*) *Grundulus bogotensis*).

**Figure 1.**
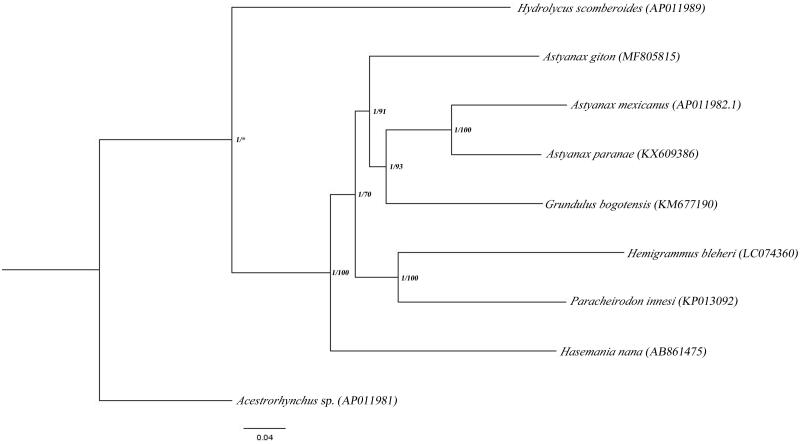
Bayesian phylogenetic tree for Characidae mitogenomes using Acestrorhynchidae as outgroup. Numbers at each node represent the posterior probability (PP) obtained in Bayesian (BI) analysis, and percentage of bootstrap values (BV) obtained using Maximum Likelihood (ML) analysis. Asterisks represent nodes that were not retrieved using ML analysis. In the ML analysis, clade consistency was verified using 1000 pseudoreplicates obtained with the ultrafast bootstrap method; BI analysis was performed using four independent chains with 10,000,000 generations and the first 25% of the generations were discarded as burn-in.

## References

[CIT0001] Bolger AM, Lohse M, Usadel B. 2014 Trimmomatic: a flexible trimmer for Illumina Sequence Data. Bioinformatics. 30:2114–2120. 24695404 10.1093/bioinformatics/btu170 PMC4103590

[CIT0002] Boore JL. 1999 Animal mitochondrial genomes. Nucleic Acids Res. 27:1767–1780. 10101183 10.1093/nar/27.8.1767 PMC148383

[CIT0003] Froese R, Pauly D. 2011 FishBase: World Wide Web electronic publication. [accessed 2017 Apr 19]. www.fishbase.org

[CIT0004] Iwasaki W, Fukunaga T, Isagozawa R, Yamada K, Maeda Y, Satoh TP, Sado T, Mabuchi K, Takeshima H, Miya M, Nishida M. 2013 MitoFish and MitoAnnotator: a mitochondrial genome database of fish with an accurate and automatic annotation pipeline. Mol Biol Evol. 30:2531–2540. 23955518 10.1093/molbev/mst141 PMC3808866

[CIT0005] Javonillo R, Malabarba LR, Weitzman SH, Burns JR. 2010 Relationships 651 among major lineages of characid fishes (Teleostei: Ostariophysi: Characiformes), 652 based on molecular sequence data. Mol Phylogenet and Evol. 54:498–511. 10.1016/j.ympev.2009.08.026 19720150

[CIT0006] Lima FCT, Malabarba LR, Buckup PA, da Silva JFP, Vari RP, Harold A, Benine R, et al 2003 Characidae, genera incertae sedis In: Reis RE, Kullander SP, Ferraris CJ, editors. Check list of the freshwater fishes of South and Central America. Porto Alegre: Edipucrs; p. 106–69.

[CIT0007] Mirande JM. 2009 Weighted parsimony phylogeny of the family Characidae. 692 (Teleostei: Characiformes). Cladistics. 25:574–613. 10.1111/j.1096-0031.2009.00262.x 34879592

